# Implementation of a cryo-electron tomography tilt-scheme optimized for high resolution subtomogram averaging

**DOI:** 10.1016/j.jsb.2016.06.007

**Published:** 2017-02

**Authors:** Wim J.H. Hagen, William Wan, John A.G. Briggs

**Affiliations:** Structural and Computational Biology Unit, European Molecular Biology Laboratory, Meyerhofstrasse 1, Heidelberg, Germany

**Keywords:** Electron tomography, Tilt-scheme, Subtomogram averaging, Cryo-electron microscopy

## Abstract

Cryo-electron tomography (cryoET) allows 3D structural information to be obtained from cells and other biological samples in their close-to-native state. In combination with subtomogram averaging, detailed structures of repeating features can be resolved. CryoET data is collected as a series of images of the sample from different tilt angles; this is performed by physically rotating the sample in the microscope between each image. The angles at which the images are collected, and the order in which they are collected, together are called the tilt-scheme. Here we describe a “dose-symmetric tilt-scheme” that begins at low tilt and then alternates between increasingly positive and negative tilts. This tilt-scheme maximizes the amount of high-resolution information maintained in the tomogram for subsequent subtomogram averaging, and may also be advantageous for other applications. We describe implementation of the tilt-scheme in combination with further data-collection refinements including setting thresholds on acceptable drift and improving focus accuracy. Requirements for microscope set-up are introduced, and a macro is provided which automates the application of the tilt-scheme within SerialEM.

## 1 Cryo-electron tomography and subtomogram averaging

In cryoET (Frank, 2008), a plunge frozen specimen is tilted using a rotating specimen stage inside a transmission electron microscope (TEM) and projection images are acquired onto a camera at discrete tilt angles. This series of tilted images can then be computationally reconstructed into a tomogram, i.e. the three-dimensional representation of the field of view. Collection of a tilt series is performed by iterative tracking, focusing and imaging steps for each tilt. Free and commercial software packages are available that allow fully automated acquisition of multiple tilt series (Mastronarde, 2005, Suloway et al., 2009, Zheng et al., 2010). Tomogram reconstruction involves tilt image alignment and 3D reconstruction. Tilt image alignment includes refinement of tilt-axis angle and tilt angles, determination of image shifts, and accounting for beam-induced sample deformation. These steps are performed computationally using software such as IMOD (Mastronarde, 1997).

The interpretable information in a cryo tomogram is limited by poor signal-to-noise ratio (SNR). However, objects present in multiple copies within the tomogram can be further analyzed by extracting them as sub-volumes. These sub-volumes can then be iteratively aligned and averaged to obtain reconstructions with improved SNR and more interpretable high-resolution features. This technique is called subtomogram averaging (for reviews see e.g. (Briggs, 2013, Förster and Hegerl, 2007, Schmid, 2011, Wan and Briggs, 2016)). Recent optimizations of data acquisition and processing have allowed the structures of protein complexes to be determined at subnanometer resolutions using subtomogram averaging (Bharat et al., 2015, Pfeffer et al., 2015, Schur et al., 2015a, Schur et al., 2015b, Schur et al., 2013), and further optimization can be expected to lead to further improvements in the attainable resolution.

## 2 Tilt-schemes for cryo-electron tomography

According to the central slice theorem, the Fourier transform (FT) of each 2D projection image in the tilt series corresponds to a slice through the 3D FT of the volume being imaged. The thickness of these Fourier slices is inversely proportional to the thickness of the object in real space. Tomographic reconstruction can therefore be envisaged as filling Fourier space with a series of slices. The slab geometry of the sample limits the range of tilts that can be collected, often to ±60°. This limited range leads to a “missing wedge” of information in Fourier space; the real space effect is a deformation of structures parallel to the axis of missing information. Fourier space is also incomplete at higher resolutions because the tilt increment is not infinitely small; the Fourier slices may not have sufficient thickness to fill Fourier space at higher resolutions. The resolution to which the information is complete is related to the number of uniformly-distributed tilt-images by the Crowther criterion m = π * D/d with number of tilt images m, particle diameter D and resolution d (Crowther et al., 1970).

In practice the distribution of information in Fourier space is complicated by three other factors. Firstly, at higher tilts the slab-shaped sample is thicker, leading to a larger number of non-elastic scattering events and a lower SNR. Secondly, frozen hydrated samples are sensitive to electron radiation damage. Higher resolution features are lost in images collected later in the tilt series due to accumulated electron dose. Thirdly, the sample may distort, bend or move as a result of exposure to the electron beam, such that the object imaged late in the tilt series is not truly identical to that imaged early in the tilt series. These factors mean that the distribution of information in Fourier space is dependent on the order and increment of angles at which the tilted images are collected, referred to as the tilt-scheme.

The ideal distribution of information in Fourier space, and therefore the preferred tilt-scheme, depends on whether the tomogram will be used for subsequent subtomogram averaging or not. If the tomogram is to be directly interpreted, it is preferable to distribute the information at the interpreted resolution as evenly as possible in Fourier space. If the tomogram is to be used for subtomogram averaging, Fourier space will be filled in the final reconstruction by averaging subtomograms that have different orientations relative to the electron beam. In this case it is usually possible to tolerate a larger missing wedge, and a larger tilt increment. Therefore, the aim when collecting data for subtomogram averaging is to maintain the maximum amount of high-resolution information while maintaining sufficiently complete low-resolution information to allow accurate 3D alignment of the subtomograms.

The most commonly used tilt-schemes fall into two groups: continuous tilt-schemes in which the tilt angle is rotated in one direction, e.g. from +60° to −60° (Fig. 1A); and bidirectional tilt-schemes, in which the tilt series acquisition is divided into two separate tilt branches, e.g. a continuous series from 0° to +60° in 3° increments is acquired first, then a second branch from −3° to −60° is acquired (Fig. 1B). Tilt increments typically range from 0.5° to 5°. Continuous tilt-schemes are mostly used for resin embedded samples. These samples suffer from mass loss when exposed to electron beam radiation and therefore deform during data acquisition. By acquiring the data in one sweep, these deformations will be gradual and can be tolerated in the tilt series alignment step. A bidirectional tilt-scheme is better suited to automated data acquisition as it does not require tilting and tracking to locate the targeted image area at high starting tilt angle before starting acquisition. A disadvantage is that the images on either side of the starting angle (0° in the above example) have different accumulated dose, and the resulting accumulated image deformations can make it difficult to align the two tilt branches (the jump-at-start problem). This error propagates into the tomogram where it is detrimental for accurate 3D alignment of the subtomograms, and for the quality of the final average.

## 3 An improved tilt-scheme for subtomogram averaging

The transfer of high-resolution information can be maximized by collecting the low-tilt images (where the sample appears thin) early in the tilt series, before radiation damage has accumulated. Our optimized tilt-scheme starts at zero degrees tilt, and moves up to the highest tilt in both tilt directions simultaneously. For example, for a 3° tilt step, the order of tilt angle acquisition is 0°, +3°, −3°, −6°, +6°, +9°, −9°, −12° … (Fig. 1C). This “dose-symmetric” tilt-scheme concentrates high-resolution information in the lower tilts where the sample is thinnest, thus providing maximum information transfer (Fig. 1D). While similar information transfer can be obtained using bidirectional tilt-schemes with a longer first branch e.g. from −21 to 60°, these schemes still suffer from the jump-at-start problem (Figs. 1D and 2).

## 4 The dose-symmetric tilt-scheme has a number of additional advantages

• The accumulated electron dose, and therefore the beam-induced sample deformation, varies smoothly across the tilt series, eliminating the jump-at-start problem (Fig. 2).
• It allows a higher total electron dose to be applied, because accumulated radiation damage and accumulated sample deformation at higher tilts is a lesser concern.
• The maximum tilt-range can be used, and high-tilt/late images can be discarded at a later stage if appropriate without losing “good signal”, or images can be exposure filtered (Grant and Grigorieff, 2015) according to the accumulated dose.
• No microscope optics other than beam-image shift and defocus are changed during tilt series acquisition, in contrast to bidirectional tilt-schemes in which magnification and illumination are changed for low magnification tracking between tilt branches. This makes the tilt-scheme ideal for use with phase plates, where optical stability is crucial (Fukuda et al., 2015).

While implementing this tilt-scheme we have also taken into account other requirements for high-resolution subtomogram averaging. Full tracking is incorporated to allow data collection at high-magnification where the field of view is small. Working at high-magnification is required to avoid resolution limitations imposed by the Nyquist limit. It can allow images to be subsequently binned for improved information transfer. A small field of view also minimizes the effect of sample distortions. Specimen drift-measurement is also incorporated. This permits a threshold for acceptable drift to be set, above which the microscope will wait before proceeding with data collection. Finally, iterative focusing (Schur et al., 2013) is implemented. In an ideal case, where the defocus can be measured directly from each image, highly accurate focusing may not be a requirement. In our experience, in many cases, especially when using CCD cameras, it is not possible to reliably determine the defocus of high-tilt images directly. In these cases we find that accurate focusing allows defocus determination by averaging power-spectra from neighbouring images in the tilt series, or allows estimation of the defocus based on the nominal focus (Schur et al., 2013)

SerialEM was used for the development of the tilt-scheme as its macro language provides a convenient way to implement automated data collection routines (Mastronarde, 2005).

## 5 Microscope set-up and calibrations

For an FEI microscope there is no need to adjust stage hardware beyond default specifications. As for all tomogram acquisition purposes, good microscope alignment and SerialEM calibrations make acquisition easier and more robust. Of particular importance for the scheme presented here are tilt axis calibration and rotation center alignment.

To minimize accumulation of radiation damage on the area of interest, tracking and focusing tasks are performed on an area displaced along the specimen stage tilt axis. For accurate autofocus results it is essential to calibrate the absolute tilt axis at the magnification used to acquire the tilt series. If it is off, the autofocus area will not be shifted exactly along the tilt axis and focus measurements do not reflect the actual focus at the acquisition area.

For subnanometer-resolution subtomogram averaging it also becomes necessary to correctly align the rotation center to minimize beam-tilt-induced coma (Glaeser et al., 2011); on a typical microscope the mechanical alignment of the stage tilt axis onto the optical axis of the microscope may be off by several micrometers. This is compensated by using a beam-image shift to move the optical axis onto the stage tilt axis. In SerialEM this beam-image shift is called “Tilt Axis Offset”; SerialEM has automated routines to measure this offset. Since this offset uses a beam-image shift, the rotation center needs to be aligned at the now shifted optical position. This can be achieved by either the Tomo Rotation Center Direct Alignment procedure of a FEI TEM, or, more accurately, by coma-free alignment. However, the FEI microscope alignment software does not allow coma–free alignment with a beam-image shift applied. To overcome this problem we have used AutoCTF, a program by FEI that allows aligning coma-free by Zemlin tableau (Zemlin et al., 1978) with a beam-image shift applied. Leginon (Suloway et al., 2009) also allows coma–free alignment with a beam-image shift applied. The AutoCTF software is currently delivered together with the FEI Volta phase-phase, but its functionality is independent of the phase-plate and we expect that similar software tools will become more widely available in due course.

## 6 Overcoming hardware limitations

The success of any tilt series acquisition depends on the movements of the specimen stage and the ability of the automated data collection routine to compensate for these. The specimen stage used to rotate the sample in the TEM is an automated goniometric stage, allowing movement of the specimen on four axes (X, Y, Z, α). Its mechanical accuracy is not sufficient to keep an image area centered and focused on the detector during acquisition of a tilt series. To keep the specimen in the field of view, any shift between consecutive tilted images in the XY plane is measured and corrected for using the TEM’s image shift coils. Similarly, any Z difference can be measured using an autofocus routine, and corrected for with an objective lens focus change (Koster et al., 1987). At high magnifications, this can only be achieved if measures are taken to optimize stage behavior as follows:

Mechanical stage backlash and elastic hysteresis of the stage vacuum seal can cause a tilt angle difference between the two tilt branches. Approaching all tilts from the same direction minimizes this difference. In our tilt-scheme, all tilts are approached from the negative side: a tilt from −3° to −6° means we first tilt to −9° and then back to −6°.

Although X and Y stage positions are not changed during tilt series acquisition, the X and Y measurement systems of the stage register specimen holder movement upon α tilt and the stage control software does not correct for this. On a Titan stage this movement is not observed on the sample, but on a Tecnai stage it is. When using Tecnai stages, moving back to the starting X and Y stage positions after each tilt angle change, reduces the total image shifts needed throughout the tilt series. On a Titan stage this is not necessary.

Stage tilt is accompanied by specimen drift; high drift causes unwanted blur in the acquired image. Most tomography acquisition software packages deal with drift using a wait-after-tilt time. We noticed that initial drift increases at higher tilt angles and in previous work using a CCD camera we used wait times of 30 s after each tilt (Schur et al., 2013). A fixed wait-after-tilt time is either too long, or is insufficient to allow stage stabilization. Here we repeatedly measure drift after each tilt until it drops below a threshold before proceeding with data collection. The threshold used is one detector pixel drift per CCD exposure or per direct detector frame.

Most autofocus routines measure beam-tilt-induced image shift and then change the objective lens focus value accordingly (Koster et al., 1987). Magnetic hysteresis of the objective lens makes this focus change non-reproducible. As described previously (Schur et al., 2013), this problem can be overcome by iterating the autofocus routine. In the macro accompanying this paper, there are two rounds of autofocus to overcome hysteresis. An alternative is to iterate until the measured defocus is within a certain user-defined target defocus range. For the most accurate autofocus results, the focus area should be as close to the acquisition area as possible to ensure autofocus is performed in the same Z plane as the acquisition. To achieve this while preventing unwanted damage to the acquisition area, we ensure that the beams of the focus and acquisition areas are adjacent but do not overlap. Further, the optical settings of Focus mode and Record mode should be exactly the same. This removes any error due to inaccuracies in parfocal alignment.

## 7 Outline of the SerialEM macro

Taking the above issues into account we have implemented our tilt-scheme using a SerialEM macro. The macro is provided in the Appendix. The macro functions as follows:

• Acquire zero-tilt image – see ZERO
• Run a loop, for each loop iteration:
  o Acquire tilt plus 1 – see TILT PLUS
  o Acquire tilt minus 1 – see TILT MINUS
  o Acquire tilt minus 2 – see TILT MINUS
  o Acquire tilt plus 2 – see TILT PLUS

### ZERO

1. Store stage position.
2. Drift stabilization.
3. Autofocus (iterate if desired).
4. Store focus value.
5. Exposure.
6. Store image shifts.
7. Get a new tracking image and store it.

### TILT PLUS

1. Tilt to angle.
2. Reset stage XY.
3. Set the previous focus value and image shift for this branch.
4. Drift stabilization and tracking.
5. Autofocus (iterate if desired).
6. Store focus value for this tilt branch.
7. Acquire tilt image.
8. Tracking-After with current and previous tilt image for this branch.
9. Store image shifts for this tilt branch.
10. Get a new tracking image and store it.

### TILT MINUS

1. Tilt to angle.
2. Backlash α tilt.
3. Reset stage XY.
4. Set the previous focus value and image shift for this branch.
5. Drift stabilization and tracking.
6. Autofocus (iterate if desired).
7. Store focus value for this tilt branch.
8. Acquire tilt image.
9. Tracking-After with current and previous tilt image for this branch.
10. Store image shifts for this tilt branch.
11. Get a new tracking image and store it.

## 8 Acquisition time and choice of microscope

Data collection with this tilt-scheme is robust and it is in routine use in our lab. The drift measurements and the autofocussing tasks for each tilt step, as well as to a lesser extent the tracking and larger changes in tilt, all contribute to increase the time required to acquire each tilt series. The amount of time is dependent on the stringency of the thresholds set for drift and focus, as well as on the behavior of the sample (whether it drifts a lot or a little). Both of these determine how often drift and focus are likely to iterate before the tilt-image is collected. Acquisition time can be reduced by removing iterative focusing, using less stringent thresholds, or by using a less conservative dose distribution by defining larger groups for each tilt branch (e.g. 0°, +1° +2° +3° +4°, −1° −2° −3° −4° etc.). Due to different stage design, a FEI Tecnai Polara microscope will show more drift-after-tilt which may increase acquisition time up to 50%. Microscopes with removable side-entry cryo-holders are unlikely to be sufficiently stable for this type of data collection.

For example, for a set of tilt series with ±66° tilt range, 3° tilt step, defocus specification of 50 nm, drift specification of 5.4 Å per second and an acquisition time of 3.3 s with 10 superresolution frames on a Gatan K2 detector on the EMBL Titan Krios, we observed that drift appeared to be high, and data collection times for a series were about 70 min. For a set of tilt series on a different sample, with ±60° tilt range, 3° tilt step, defocus specification of 50 nm, drift specification of 8.7 Å per second and an acquisition time of 1.55 s with 10 superresolution frames, where drift appeared to be low, we observed data collection times of about 45 min, which is comparable to alternative schemes.

The tilt-scheme as implemented allows sufficiently accurate image tracking to reliably work at high-magnifications where the field of view is as small as 200 nm. Depending on the condition of the stage mechanics however, a lower magnification might be needed for tracking,

A current disadvantage of the tilt-scheme macro is that it does not detect blocked images and abort tomogram data collection. We hope that in the future the scheme will be fully incorporated into SerialEM, and this limitation will no longer apply.

## 9 Summary

The amount of high-resolution information in a tomogram is greatest if all low-tilt images are collected early in the tilt series, and all high-tilt images late in the tilt series. Optimizing the amount of high-resolution information is particularly important for subtomogram averaging. Here we have developed a tilt-scheme that allows data collection in this manner, and implemented it in the form of a SerialEM macro that other labs can make use of. We now use this tilt-scheme for routine data collection in our lab. Prior to processing of data collected with this tilt-scheme, high-tilt images can be discarded in the case that they appear too thick or overexposed to provide useful information. We, however, prefer to filter each image according to its accumulated dose, analogous to the approach described for single-particle movie processing in (Grant and Grigorieff, 2015). We believe that the tilt-scheme described here will become established as the method-of-choice for subtomogram averaging.

## Figures and Tables

**Figure:**
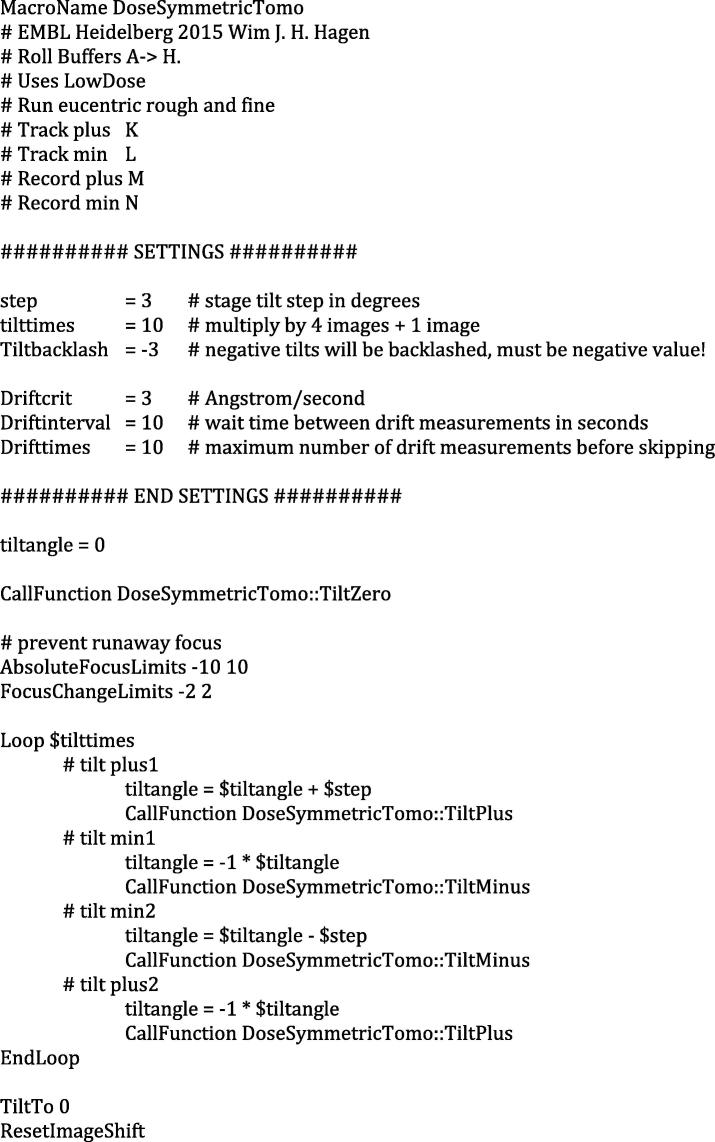


**Figure:**
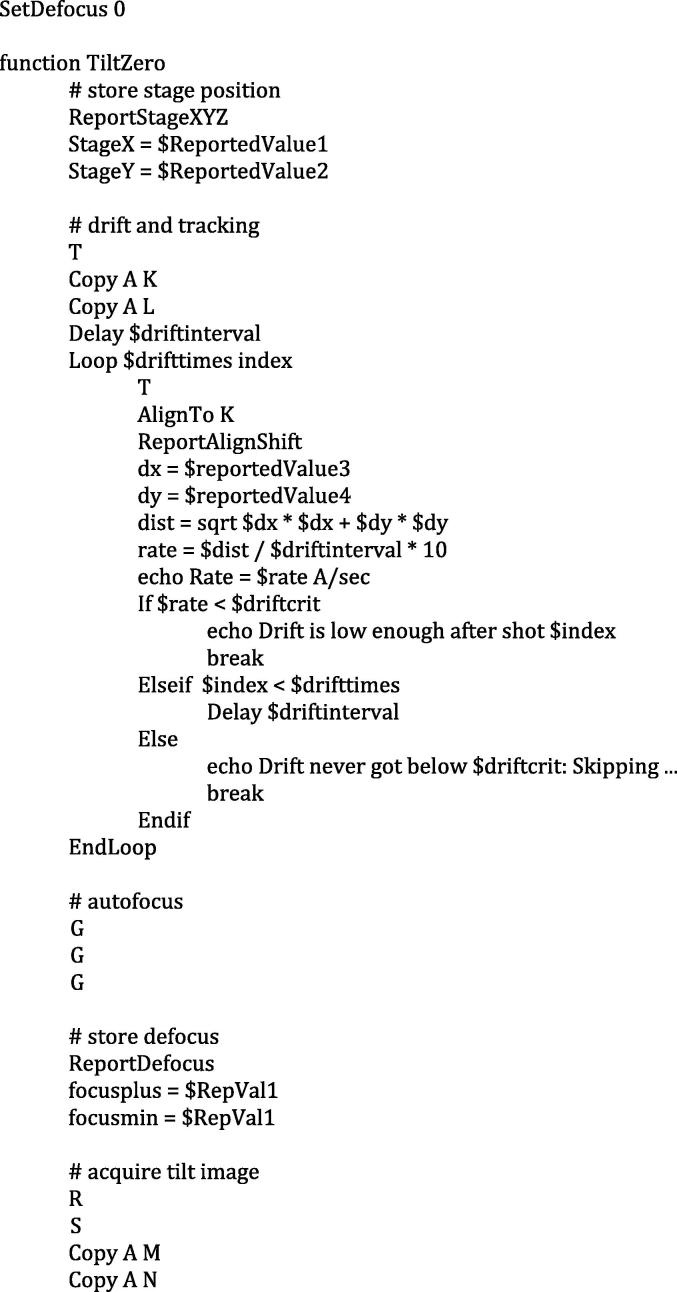


**Figure:**
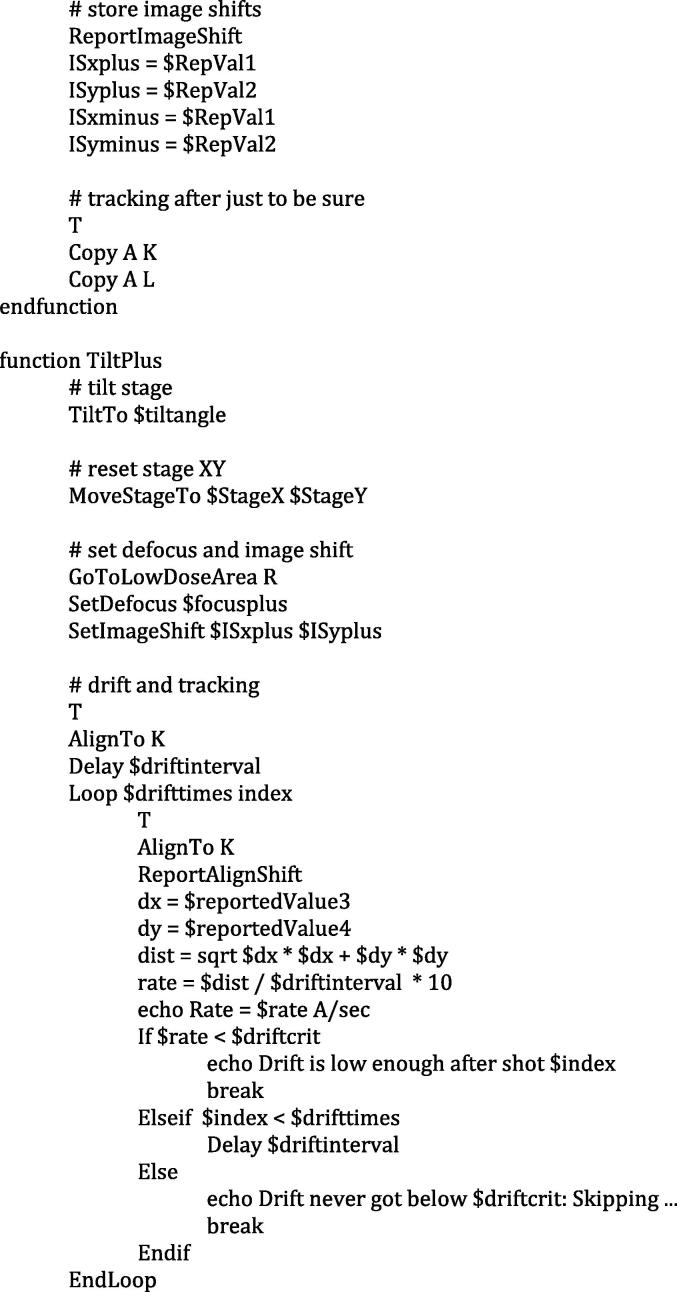


**Figure:**
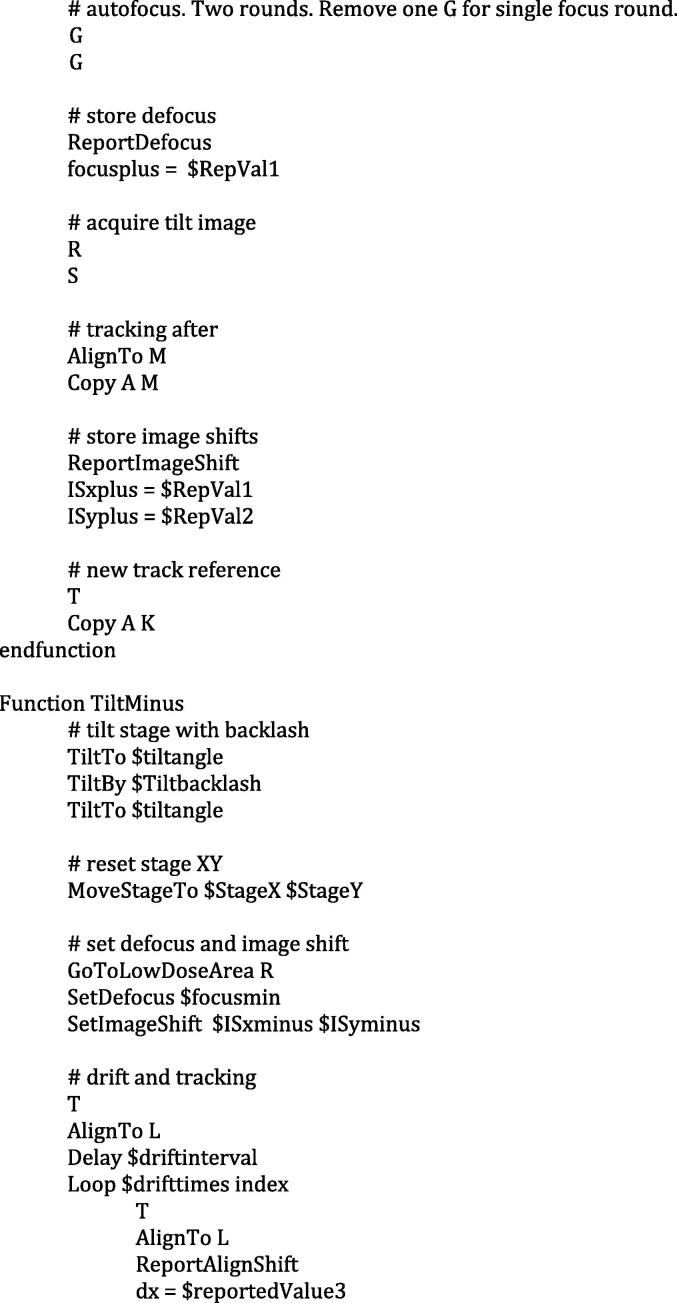


**Figure:**
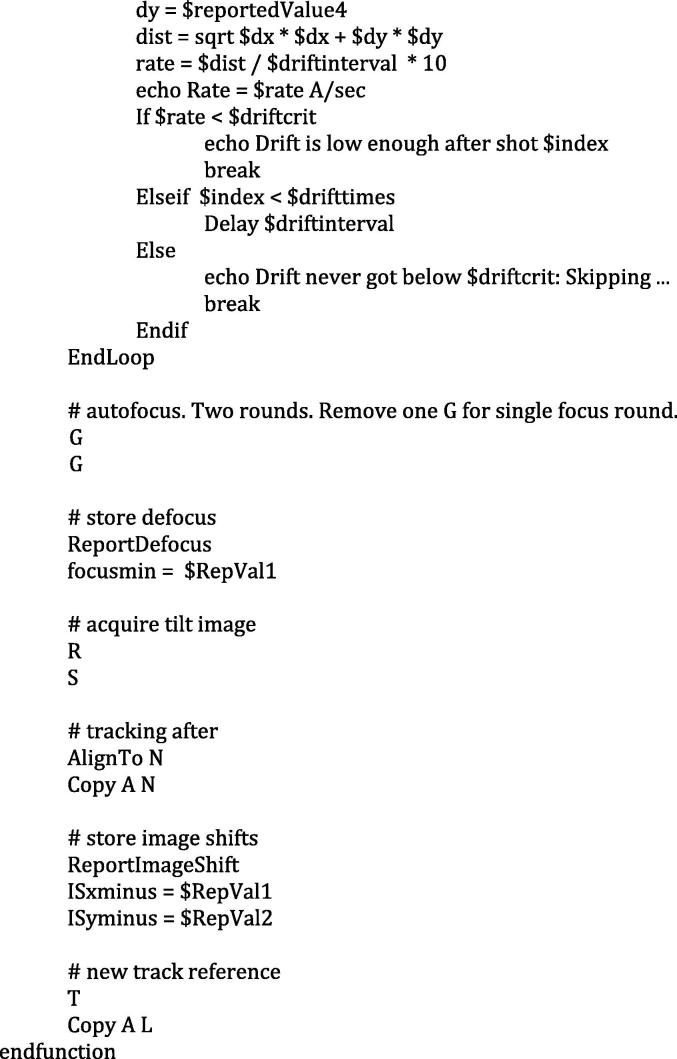


**Fig. 1:**
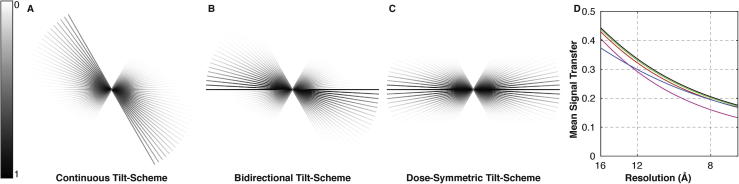
Schematic showing information transfer for (A) continuous, (B) bidirectional and (C) dose-symmetric tilt-schemes. Tilts are shown from −60° to +60° in 3° increments for a total of 41 tilts. Grey values correspond to the information transfer at each tilt according to the color map shown on the left. The reduction of information transfer at high-tilts due to the increased apparent thickness of the sample is simulated by multiplication with the cosine of the tilt angle. The loss of high-resolution information due to accumulated electron dose is simulated by multiplication by low pass filters according to the measurements described in (Grant and Grigorieff, 2015) assuming constant exposure times. The dose-symmetric tilt-scheme shows optimized, near-symmetric information transfer. (D) Plot of mean signal transfer for different tilt-schemes: continuous (magenta); bidirectional starting at 0° (red); bidirectional starting at −21° (green); bidirectional starting at −21° in the case that the second branch is deleted before averaging to reduce impact of the jump-at-start problem (blue); dose-symmetric (black). Mean signal transfers are calculated as the mean of the signal transfer for all tilts within the tomogram.

**Fig. 2:**
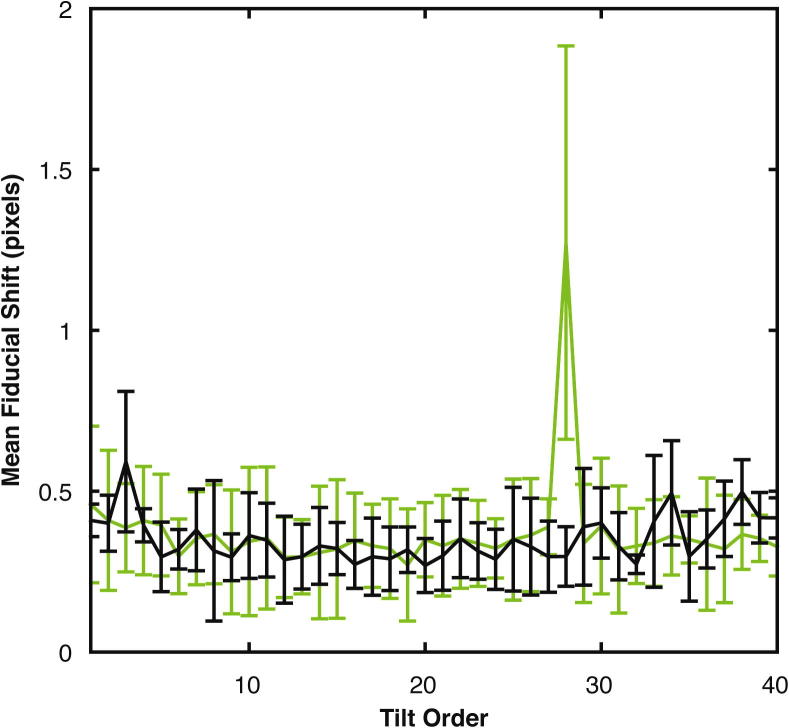
Alignment errors in tilt series collected using different tilt-schemes. Five tomograms were acquired from 3 grids using either bidirectional starting at −21° (green) or dose-symmetric (black) tilt-schemes, during the same data collection sessions. Tilt series were aligned, and the mean fiducial shifts parallel to the tilt axis between each tilt and the adjacent tilt in the positive direction were calculated (e.g. between −60° and −57°, −57° and −54°, …). This is equivalent to the slope of the “marker tilt line” at each tilt. These were then averaged over all tomograms and plotted with respect to tilt-image collection order. Error bars are the standard deviation of the mean fiducial shifts between each tomogram. The mean fiducial shifts are similar for bidirectional and dose-symmetric tilt series, with the exception of a large mean fiducial shift in the bidirectional tilt-scheme between −21° and −24°, i.e. the point where the two branches are joined. This results from the jump-at-start problem, where the second branch of the tilt series is collected after the sample has a larger accumulated dose with resulting specimen deformation.
